# Postharvest Analysis of Lowland Transgenic Tomato Fruits Harboring hpRNAi-*ACO1* Construct

**DOI:** 10.1100/2012/439870

**Published:** 2012-07-31

**Authors:** Bita Behboodian, Zainon Mohd Ali, Ismanizan Ismail, Zamri Zainal

**Affiliations:** ^1^School of Biosciences and Biotechnology, Faculty Science and Technology, UKM, Selangor, 43600 Bangi, Malaysia; ^2^Institute of System Biology, UKM, Selangor, 43600 Bangi, Malaysia

## Abstract

The plant hormone, ethylene, is an important regulator which involved in regulating fruit ripening and flower senescence. In this study, RNA interference (RNAi) technology was employed to silence the genes involved in ethylene biosynthetic pathway. This was achieved by blocking the expression of specific gene encoding the ACC oxidase. Initially, cDNA corresponding to *ACO1* of lowland tomato cultivar (MT1), which has high identity with *ACO1* of *Solanum lycopersicum* in GenBank, was cloned through RT-PCR. Using a partial coding region of *ACO1*, one hpRNAi transformation vector was constructed and expressed ectopically under the 35S promoter. Results showed that transgenic lines harboring the hpRNA-*ACO1* construct had lower ethylene production and a longer shelf life of 32 days as compared to 10 days for wild-type fruits. Changes in cell wall degrading enzyme activities were also investigated in cases where the transgenic fruits exhibited reduced rates of firmness loss, which can be associated with a decrease in pectin methylesterase (PME) and polygalacturonase (PG) activities. However, no significant change was detected in both transgenic and wild-type fruits in terms of **β**-galactosidase (**β**-Gal) activity and levels of total soluble solid, titratable acid and ascorbic acid.

## 1. Introduction

Fruit constitutes an important part of daily diet, thus contributing to its demand in local and worldwide markets. Due to its ever-growing market, the maintenance of certain fruit traits, including nutritional value, flavor, processing qualities, and shelf-life is desirable [1]. This has encouraged many molecular biologists to study the complexity of fruit ripening so that the plants can be genetically manipulated to enhance the edible quality of the fruits in terms of color, flavor, and aroma.

The inhibition of fruit ripening is achieved by reducing ethylene production [2], such as by downregulating genes encoding key enzymes in the ethylene biosynthetic pathway. For example, antisense constructs have been expressed to reduce the activity of ACC synthase [3] and ACC oxidase [4]. Antisense suppression of ACC oxidase expression has been used to reduce ethylene biosynthesis in melon [5], broccoli [6], and the ornamental flower, *Torenia fournieri *[7]. Additionally, Peters et al. (1999) [8] generated transgenic melon lines expressing the antisense ACC oxidase gene from apple. An alternative approach is the diversion of metabolic flux away from ethylene synthesis by overexpressing enzymes involved in ACC degradation. This was achieved by using constructs encoding the enzymes ACC deaminase [9] and *S*-adenosyl methionine (SAM) hydrolase [10].

Scientific advancement in gene silencing has rendered more effective and reliable methods for the use of RNA interference (RNAi). The RNAi concept lies in its sequence-specific RNA degradation principle, where the most efficient method to silence an endogenous gene in plants is through a hairpin RNA (hpRNA). This RNA consists of an inverted repeat of a gene sequence fragment and is separated by an intron to increase the frequency of silencing [11]. Helliwell et al. (2002) [12] began RNAi research to test the effectiveness of the pHellsgate vector for gene silencing in arabidopsis. Several successful studies using RNAi to delay fruit ripening have been reported, and the ACC oxidase gene is successfully silenced in tomatoes [13]. Additional research indicates that suppressing the transcription of the endo-p-mannanase (LeMan4a) gene using an RNAi strategy results in the reduction in endo-p-mannanase activity in transgenic tomato fruits and, therefore, results in firmer fruits as compared to the wildtype fruits, which turned orange earlier [14]. [15] also showed enhanced tomato shelf life and reduced rate of softening in approximately 30 days by suppressing two ripening-specific N-glycoprotein modifying enzymes: alpha mannosidase (alpha-Man) and beta-D-N-acetylhexosaminidase (beta- Hex).

Until today, many RNAi studies on *Solanum lycopersicum* temperate varieties have been carried out. Nonetheless, little information on lowland varieties is available. Therefore, we were interested in studying the lowland *Solanum lycopersicum* cv. MT1 variety, which was developed by Malaysia Agriculture Research and Development Institute (MARDI) through crossing the CL555-10 lines with the local white variety. This variety bears smaller-sized fruits with shorter shelf life than the temperate varieties. In this study, a partial cDNA corresponding to *ACO1* (ACC oxidase) from lowland tomato was cloned, and several transgenic lines were produced through RNAi technology. Here, we describe the biochemical and physiological aspects of the transgenic RNAiACO1 fruits.

## 2. Material and Methods

### 2.1. Cloning of Partial ACO1 cDNA and hpRNAi Transformation Vector Construction

Total RNA was isolated from wounded leaves. The method used for RNA extraction was the method of Lopez-Gomez (1992) with slight modification [16]. Extracted RNA was subjected to the first-strand cDNA synthesis. Degenerate primers DPACOF forward primer (5′ GAY TAY AAR AAR TGY ATG GAR CAR 3′) and DPACOR reverse primer (5′ ACY TTN TGN CCR AAR TTY GG 3′) were designed from the conserved region of aligned amino acid sequence of ACC-oxidase and used in subsequent RT-PCR reactions. The amplicons were then cloned into the pGEMT vector (Promega) and sequenced.

**Figure 1:**
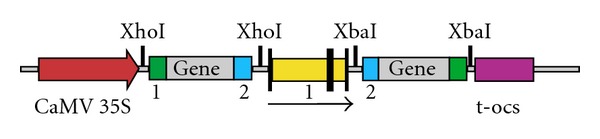
Schematic diagram of the binary vector constructed for transformation of *ACO1* gene in transgenic tomato plants.

For RNAi construction, the cloning method was based on site-specific recombination. A 368 bp fragment was amplified from pGEMT vector using gene-specific primers which linked to the att-B sequence (attB1-ACO-F 5′GGGGACAAGTTTGTACAAAAAAGCAGGCTCGTGTCCGAAGCCTGATCT3′, attB2-ACO-R 5′-GGGGACCACTTTGTACAAGAAAGCTGGGTCCAGCAATTCTGGTGCTG3′). The attB-PCR product was purified, and BP recombination reaction with this product was performed with pDONR to produce an entry clone according to manufacturer's protocol [17]. Finally, a pDONR to pHellsgate recombination reaction was performed using LR Clonase. An aliquot of LR reactions was used for transformation into *E. coli* DH_5_ *α*, and the recombinant vector was designated pHsgt-RI (Figure 1).

### 2.2. Generation of Transgenic Tomato Plants

The recombinant plasmid was transferred into *Agrobacterium tumefaciens* strain LBA 4404 using the freeze-thaw method and subsequently used for tomato transformation. Tomatoes (*Solanum  lycopersicon* cv. MT1) were transformed according to Ling et al. (1998) [18]. Cotyledon and hypocotyls explants of 8-day-old seedlings were used as explants source for cocultivation with *A. tumefaciens*. After cocultivation, explants were transferred onto selection medium Murashige-Skoog salts containing 2.0 mg L^−1^ zeatin, 0.1 mg L^−1^ indole-3-acetic acid (IAA) supplemented with 100 mg L^−1^ kanamycin and 500 mg L^−1^ cefotaxim. Transformed calluses were excised and transferred into regeneration medium. Upon regeneration, plantlets were rooted with antibiotic selection. Rooted plants were transplanted in vermiculite, and after 10–15 days, they were shifted to a glasshouse. The established plants were designated as T0-generation plants. 

### 2.3. PCR, Southern and Northern Blot Analyses of Transformed Plants

For the confirmation of putative transformants, genomic DNA was isolated from leaves of putative transgenic and wildtype plants using the CTAB method [19]. PCR was performed using a set of primers that were designed from CaMV 35S promoter sequence until the end of ACO sequence: 35S-F: 5′ CCC ACG AGG AGC ATC GTG GAA3′ and attB2-ACO-R: 5′GGG GAC CAC TTG TAC AAG AAA GCT GGG TCC AGC AAT TCT GGT GCT G3′. 

DNA (20 *μ*g) was digested overnight with *Hin*dIII and separated on a 0.8% TAE agarose gel. The DNA in the gel was blotted onto nylon membranes (Hybond N+, Amersham Biosciences) by capillary blotting and cross-linked by UV irradiation. Membrane was hybridized with a 351 bp DIG-labeled probe derived from the purified fragment encompassing the CaMV 35S promoters. Hybridization and signal detection procedures were carried out according to Sambrook et al. (1989) [20]. Total RNA was isolated from T1 transgenic tomato fruits using TriReagent [21]. Twenty *μ*g of total RNA was fractionated on a 1.2% agarose gel containing formaldehyde, blotted onto a nylon membrane and covalently cross-linked by exposing to UV light. The transferred RNA was hybridized with a 368 bp DIG-labeled probe, prepared from the corresponding cDNA. After hybridization, the membrane was washed with the first washing buffer (2 × SSC, 0.1% SDS) for 10 min at room temperature, with the second (1 × SSC, 0.1% SDS) for 15 min at room temperature, and with the third washing buffer (0.1 × SSC, 0.1% SDS) for 15 min at 65°C. Presence of the transcript was detected by color development reaction.

### 2.4. Postharvest Study

Tomato (Solanum *lycopersicom *cv. MT1) fruit used in this experiment was harvested from the MBT Experimental Plot at the Universiti Kebangsaan, Malaysia, in June 2009. Fruit of T1 generation were harvested at mature green stage and left to ripen at ambient temperature (25°C) on the open laboratory bench. Sampling was done on an alternate day from each group (data for transgenic plants are represented every four days).

### 2.5. Determination of Ethylene Production and Respiration Rate

Ethylene production from ripe fruit was measured by enclosing four-to-six pieces of fruit in containers (355 mL) and allowing ethylene and carbon dioxide (CO_2_) to accumulate for 2 h. Gas samples were collected from the headspace to determine ethylene and CO_2_ concentrations. Samples were analyzed for ethylene using a Perkin Elmer gas-chromatograph (Clarus 500, USA), flame ionization detector, and a stainless steel column packed with alumina. The carrier gases consist of pure nitrogen. For CO_2_ measurements, the gas chromatograph equipped with a thermal conductivity detector and helium gas as the carrier gas. The temperature of the injector, detector and oven was 100, 220, and 50°C, respectively. 

### 2.6. Physicochemical Analysis

Tomato skin color was evaluated using reflectance meter (Minolta Chromameter, Japan) and recorded as numerical values of a*, indicating a color range from green to red, whose values are −60 to +60. Fruits were harvested in mature green stage (MG), in a* value of around −17. Breaker (BR) fruits had a* value of about −12, orange (OR) fruits a value of 20. Measurement was continued until the fruits reached the red ripe (RR) stage, with a value of 37 [22]. For determining weight loss, fruits were weighed individually every sampling day on an electronic balance (Scaltex, Germany). Loss in weight was calculated, and results were expressed as percent weight loss. Soluble solid content was determined each sampling day from the tomato fruit juice obtained using Palette Digital Refractometer (model DBX-55, Atago, Japan). The results were expressed as Brix, and all of the readings were taken at room temperature (25°C). Total titratable acidity (TA) was measured using 5 g of tomato juice diluted with 20 mL of distilled water by titration with 0.1N NaOH to an endpoint of pH 8.1 using an automatic titrimeter (model 719 S Titrino, Metrohm Ion Analysis Ltd., Switzerland). Total acidity was determined using citric acid equivalent mass because of the acid dominance in tomato. Results were expressed as percentage of grams of citric acid equivalent per 100 g fresh weight. Ascorbic acid contents were determined by using the 2,6-dichlorophenol indophenols titration (DCPIP) dye method according to Ranggana (1977) [23].

### 2.7. Firmness and Cell Wall Degrading Enzymes Extraction and Assay

Fruit firmness was determined by measuring the amount of force (*N*) to puncture a hole through the fruit. Three measurements were performed along the equatorial region of each fruit using a Texture Analyzer Machine, TA-XT Plus (Stable Micro System, England).

To start enzymes extraction and assay, tomato fruits (10 g) were homogenized in 10 mL of 0.1 M citrate buffer (pH 4.6) containing 1 M NaCl, 13 mM EDTA, 10 mM 2-mercaptoethanol and 2% (w/v) polyvinylpyrrolidone (PVP-40). The homogenate was then centrifuged (Sorvall RC-5B Superspeed) at 29,000 g for 20 minutes at 4°C. Supernatants were recovered by filtering through a double layer of nylon cloth, and the volume was determined for the enzyme assay. Polygalacturonase (PG), pectin methylesterase (PME), and *β*-galactosidase (*β*-gal) were assayed [24].

### 2.8. Statistical Analysis

The experiments were conducted using a completely randomized design (CRD) with four replicates. Analysis of variance (ANOVA) was carried out using the MSTAT-C software [25], while the least significant difference (LSD) test was used to compare differences between treatments at 95% confidence level of each variable [26].

## 3. Result and Discussion

**Figure 2:**
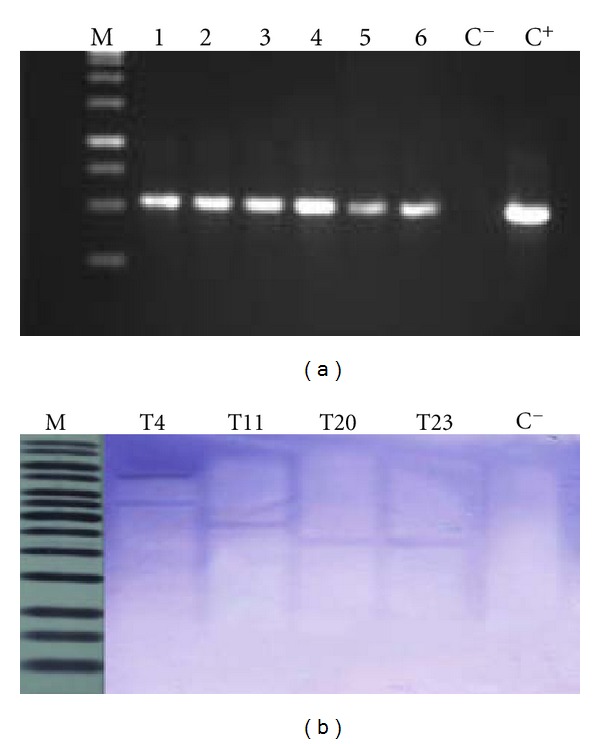
(a) Detection of transgenes in regenerated tomato plants by PCR. Lane* M*, 1 kb marker; lanes* 1–6* transgenic plants; lane* C* ^−^, negative control (nontransformed plants); lane* C* ^+^, positive control (plasmid pHsgt-RI), (b) Southern hybridization analysis of tomato fruits.

Screening of transgenic tomatoes was done using genomic DNA isolated from kanamycin resistant lines as templates. Positive bands with an expected size of 552 bp were observed in 29 out of 120 putative transgenic lines produced.  Figure 2(a) shows six of the selected transgenic lines.

**Figure 3:**
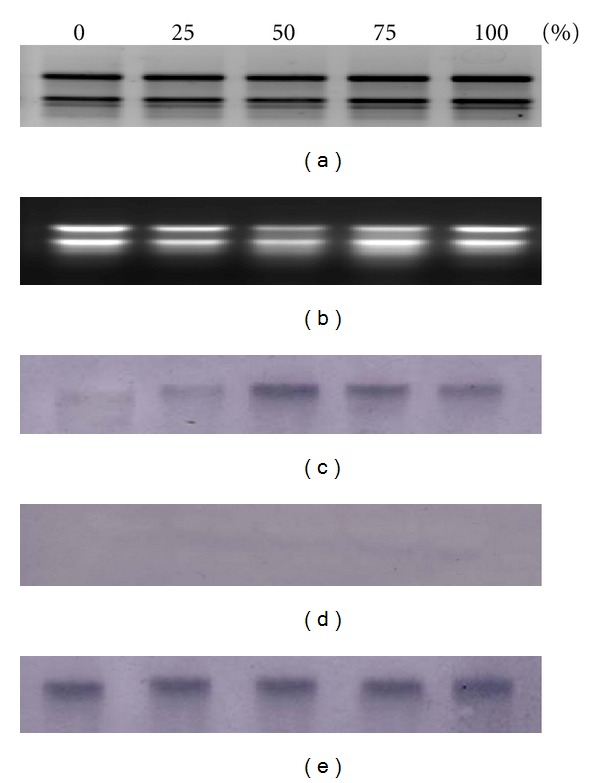
RNA expression patterns through tomato fruit ripening as determined by Northern blot. Wells 1–5 are tomatoes at ripening stages: 0, 25, 50, 75, and 100%. Total RNA extracted from (a) wildtype, (b) RNAi transgenic fruits. RNA expression patterns in (c) wildtype, (d) RNAi fruits, and (e) actin gene as an internal control.

To confirm the integration of the transgene, Southern blots were performed on genomic DNA digested with *Hind*III.  Figure 2(b) indicates that, in line T4, two bands were prominent, suggesting that two copy numbers of the transgene are integrated, whereas only a single copy gene was observed in lines T11, T20, and T23. To determine the expression levels of endogenous *ACO*, total RNA from different stages of tomato fruit ripening (0, 25, 50, 75, and 100% ripening) were isolated. Results from northern blots show that *ACO* transcript was first detected in mature green fruits of control tomato fruits at a very low level, which is considered 0% ripening. The transcript increased gradually and reached its peak at 50% ripening, followed by slightly reduced levels toward 100% ripening. This observation is contradictory with transgenic RNAi lines, where almost no signal was detected. From this result, it is suggested that *ACO1* gene was successfully downregulated using the RNAi construct (Figure 3).

### 3.1. Ethylene Production and Respiration

In all transgenic lines, there were no significant differences in the time taken for fruit to develop from anthesis to breaker stage. Fruits were harvested at the mature green stage. Transgenic tomatoes showed prolonged shelf life of more than 32 days, while it took 10 days to reach full ripeness in wildtype tomatoes. Ethylene production varied between transgenic lines (data not shown). The best lines, T11 and T23, which showed more than 75% reduction in ethylene production, were selected for further analysis.

**Figure 4:**
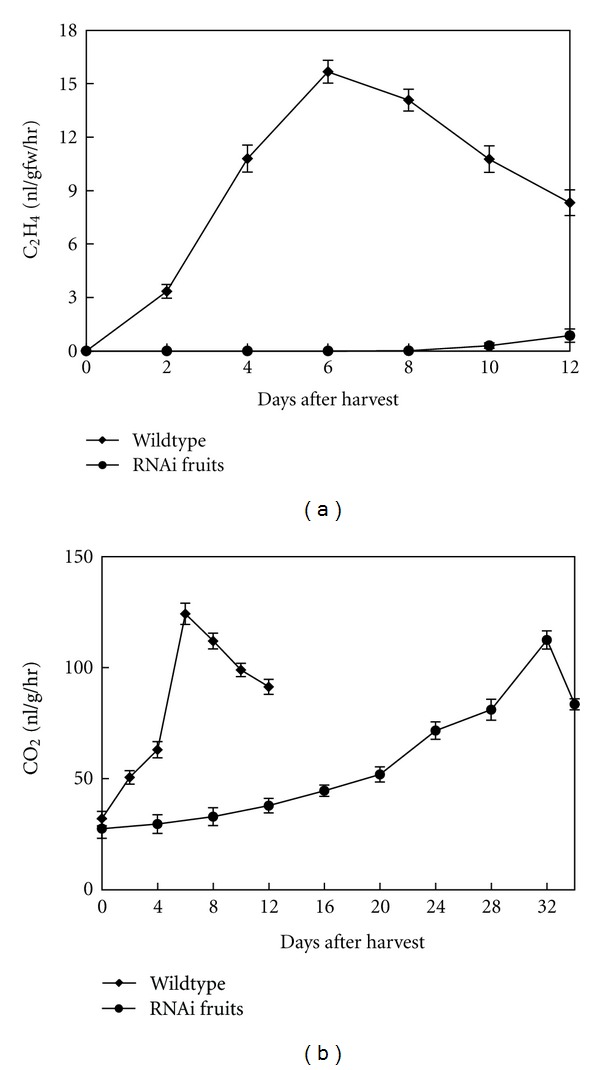
(a) Ethylene production and (b) respiration rate in tomato fruits during ripening.Each value is the mean ± SE of 4 containers.

Ethylene production and respiration rates of tomato transgenic fruits were affected by *ACO1* suppression (Figure 4(a)). Ethylene levels measured during ripening. Transgenic fruits had a significantly (*P* < 0.05) lower rate of ethylene production than wildtype fruits during storage period. A rapid increase of ethylene in wildtype fruits occurred at the first 6 days after harvest and declined in subsequent phase of ripening. In transgenic RNAi fruits, no ethylene was detected in the first 8 days, and a delay in attainment of the ethylene evolution peak to day 28 was observed. In comparison with wildtype, the peak observed in RNAi fruits was with 3.4 fold reduction.

The rate of CO_2_ production during ripening of wildtype fruit reached a peak at day six after harvest (124.2 ± 4.8 nL/g/hr) and then declined, while in RNAi fruits, respiratory peak was delayed to day 32, and the highest level (112.4 ± 3.1 nL/gr/hr) was significantly less than that observed in control fruit (Figure 4(b)). Significant differences were found between wildtype and transgenic fruit in the temporal aspect of CO_2_ accumulation. In this study, decreasing ethylene production resulted in reduced fruit metabolism in transgenic fruit, which leads to less CO_2_ production during postharvest life and delayed peak.

Ethylene is known to play an utmost important role in regulating fruits ripening and its development especially in climacteric fruits by coordinately inducing the expression of large numbers of genes which finally contribute to ripening [27]. The inhibition of ripening has been achieved in fruits by reducing ethylene production. Different approaches have been used on tomato plants. One approach involves the downregulation of genes encoding key enzymes in the ethylene biosynthetic pathway. By antisense inhibition of ACC oxidase [28, 29], or RNAi inhibition of *ACO* [13] or ACC synthase antisense [3], it has been possible to delay tomato fruit ripening. Transgenic tomato plants were produced which expressed antisense copies of an apple fruit ACC-oxidase RNA. In the fruit of the primary transformants, ethylene production was reduced by over 95% in one of the lines assessed [30]. In addition, virus-induced gene silencing (VIGS) technique has been successfully applied on tomato *ACS* gene too. VIGS markedly postponed the onset of the ethylene burst [31]. As with antisense *LeACS2* tomato fruit [32], ethylene synthesis remained at a lower level in VIGS tomatoes than in controls with *LeACS2 *silencing. Present results are in total agreement with those reports. The reduction of ethylene levels obtained by silencing of ACC oxidase possibly implies that less ethylene molecules are available to bind to receptors, so that fruit ripening is altered. Since genetic manipulation applied here does not block ethylene production altogether, it is possible that the traces of ethylene produced by the fruit were enough to induce ripening albeit at a lower rate. 

The results also show a strong correlation between ethylene evolution from fruits and *ACO1 *mRNA expression level (Figure 3). As ethylene induces the expression of *ACO1* in tomato [33], however, the signals were detected in 75% and 100% transgenic fruits at a very low basal level.

Besides ethylene, the rate of CO_2_ production, during ripening of both wildtype and transgenic fruits, exhibited a typical climacteric pattern of respiration. Rate of respiration is often a good index for the storage life of fresh fruits and vegetables; that is, the higher the rate, the shorter the life, and the lower the rate, the longer the life. Accordingly, in this study, tomato spoiled in a shorter period under conditions in which the fruit respired more rapidly. This fact was especially apparent in the case of wildtype tomato fruits (Figure 4(b)).

### 3.2. Physicochemical Analysis

#### 3.2.1. Color Development

It is evident from the data that a* values of tomato color affected by RNAi *ACO1* silencing technique during storage and the color development was significantly slower in transgenic fruits (Table 1).

As the storage continued, the green color began to fade, more in the wildtype fruits as seen by their faster increases in a* values. In these fruits, BR stage observed in day 2 (−12.88), OR stage in day 6 (20.95) and RR stage in day 10 with 37.57 in a* value. These stages happened in days 8 and 20 after harvest in RNAi fruits (Table 1). The differences observed between wildtype and transgenic fruits in any particular ripening stage (MG, BR, OR, and RR) were not statistically significant (*P* > 0.05).

In this study, development of the skin color was delayed in transgenic fruits, and these results suggest that the activity of enzymes involved in these processes is somehow associated with ethylene. The retardation of color development in transgenic tomato fruit could be attributed to the low ethylene production and delayed increment in ethylene production to reach the threshold concentration for indication of color development.

#### 3.2.2. Weight Loss

**Figure 5:**
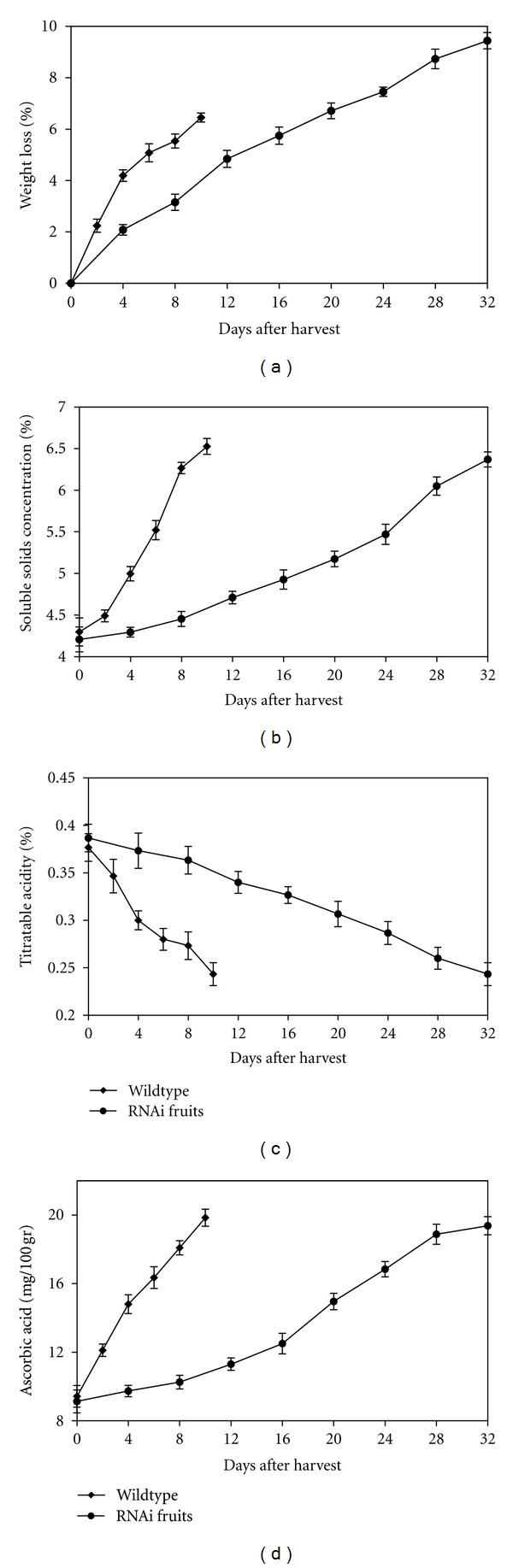
Changes in (a) weight loss, (b) soluble solids concentration, (c) titratable acidity, and (d) ascorbic acid of transgenic and wildtype tomato fruits during ripening. Each value is the mean of four replicates. The vertical bars represent the standard errors.

Figure 5(a) shows that the weight loss increased in both wildtype and transgenic fruits during storage time. At the ripe red stage, the wildtype fruits had only lost 6.4% of their weights, while the RNAi fruits lost 9.4%. The postharvest weight loss of fruit is mainly the loss of water due to environmental conditions. In this study, the weight loss was significantly higher in the RNAi fruits at every stage of ripening. As the environmental conditions were the same, the observed significant difference is due to the long storage time. These differences could be attributed to the fact that water vapour from fruit transpiration that usually occurs during storage [34]. Storage duration and storage temperature have significant effects on weight loss [35].

#### 3.2.3. Soluble Solids Concentration (SSC)

The mature green fruit of wildtype plants had soluble solid content (SSC) of 4.29%. These values reached 6.52% toward the end of storage (Figure 5(b)). For RNAi-ACO fruits, the increment of SSC content was delayed. SSC content started to increase notably from day 8 to day 32, with a value of 4.45 to 6.37%, respectively. No significant differences were observed at the end of the storage time in both fruits.

The soluble solids include organic acids, reducing sugars, and other constituents of the fruit sap affecting the % SSC. Sugars and organic acids develop during the ripening process and tend to increase with the import of sugar from the plant and from mobilization of the starch reserves in the fruit itself [36]. Under *ACO1* suppressed condition, retardation in the respiratory activity also seemed to retard the synthesis and use of the metabolites resulting in the smaller amounts of SSC present in the tissues, thereby reducing the catabolic activity and consequently delaying the ripening and prolonging the postharvest life of the fruit. The slower respiration also slows down the synthesis and use of metabolites resulting in lower SSC due to the slower change from carbohydrates to sugars [37].

#### 3.2.4. Total Titratable Acidity (TA)

Results obtained from our study show that TA decreased with the storage time in both wildtype and transgenic fruit. The initial total acid contents at day zero were in the range of 0.37% for the control to 0.38% for RNAi fruit (Figure 5(c)). After 10 days, the TA level in wildtype decreased to 0.24% and closely followed by the transgenic fruit (0.23%) after 32 days. There are no statistically significant differences between these two samples.

In general, organic acid levels can be used as an indicator for postharvest quality. Acid levels decline during ripening, presumably due to its consumption during respiration. The titratable acidity decreases throughout the fruit development until full maturity. In tomato, citric acid is a major organic acid, and its level decreases during ripening [38]. The decrease in total acidity in tomato during ripening is probably due to the decrease in citric acid. Our results showed that RNAi fruits took a longer time to reach the same levels of citric acid in comparison with the wildtype fruits. Thus, it is suggested that RNAi ACO fruits exhibited slow respiratory metabolism and, therefore, decreased consumption of the acids. It is also indicated from the acidity results that delayed ripening by *ACO1* gene silencing did not affect the quality of the fruits. At the end of storage, the contents of titratable acids in wildtype and transgenic tomato fruit were at similar levels.

#### 3.2.5. Ascorbic Acid Content

The level of ascorbic acid was also measured (Figure 5(d)). The ascorbic acid in wildtype fruits increased to the maximum of 19.84 mg 100 g^−1^ after day 10, while in transgenic fruit it took 32 days to reach to the maximum 19.37 mg 100 g^−1^. However, their differences were not statistically significant, and RNAi plants only showed delayed increases in ascorbic acid content without altering its level.

In present research, the slower increase in ascorbic acid in transgenic fruits suggests that the *ACO1* gene silencing methods slowed down but did not prevent the synthesis of ascorbic acid during ripening. It is proposed that the recorded delay in the synthesis of ascorbic acid may be due to slowing down the respiration and other metabolic activities which might have slowed the ascorbic acid synthesis. Similar slowing down of the increase in ascorbic acid during ripening has been reported with high CO_2_ storage atmospheres for tomatoes [39] and coating tomato fruits with 5 to 20% of gum Arabic [40].

### 3.3. Firmness Changes and Polygalacturonase, Pectin Methylesterase and *β*-Galactosidase Activities

#### 3.3.1. Firmness

**Figure 6:**
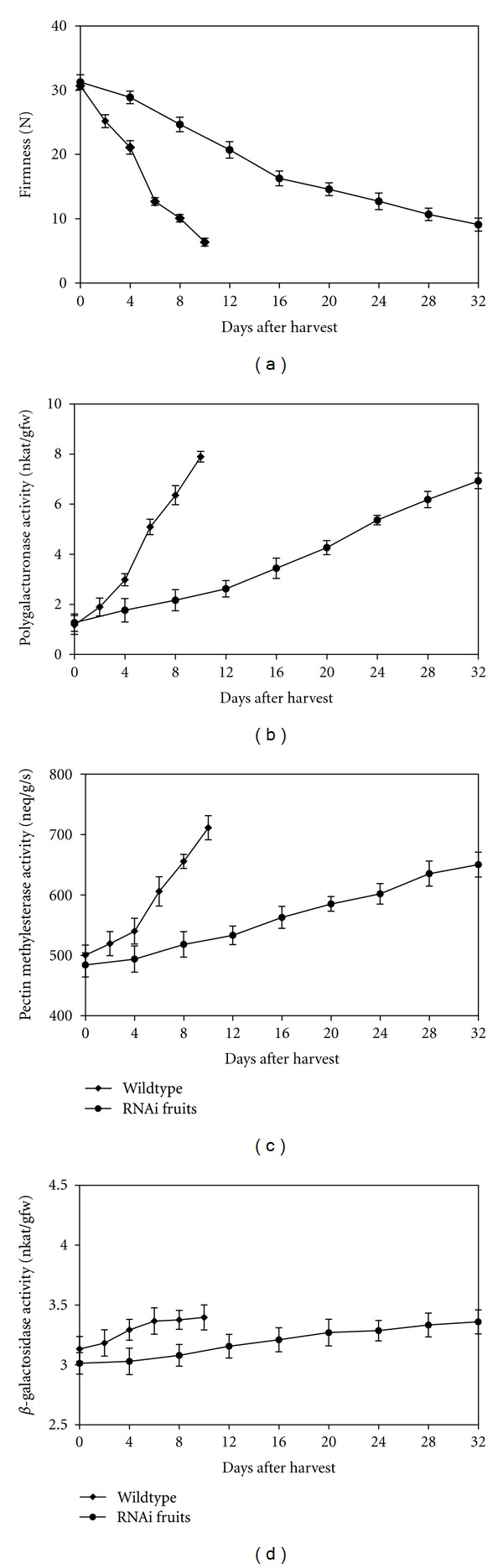
Changes in firmness (a), polygalacturonase (b), pectin methylesterase (c), and *β*-galactosidase activity (d) of transgenic and wildtype tomato fruits during ripening. Each value is the mean of four replicates. The vertical bars represent the standard errors.

Firmness decreased with increased storage period at a slower rate in RNAi fruit. In the beginning of storage, all the fruits were very firm (firmness from 35.89 N to 36.81 N), but for wildtype fruit, a large reduction in firmness occurred between day 2 and day 8; however, RNAi fruits lost their firmness slowly to 13.66 N at the end of the storage period (Figure 6(a)). There was a significant (*P* ≤ 0.05) difference in the firmness between wildtype and RNAi fruit, at fully ripe stage (12.15 and 13.66 N in day 10 and day 32, resp.).

The softening was greatly reduced with *ACO1* gene silencing methods. The wildtype fruits lost their textural integrity faster than the transgenic fruits. The maintenance of firmness in transgenic fruits is suggested to be due to their higher reducing respiration and other ripening processes during storage. In these fruits, the firmness started to decrease, while the ethylene level was still very low (Figure 4(a)). This suggests that even a small amount of ethylene is sufficient to induce fruit softening. When ethylene production is avoided by suppression of *ACO1*, the increase in ethylene production was significantly reduced, and softening of the fruit delayed. This suggests that softening is dependent on ethylene. The observed decrease in firmness levels in day 8 in transgenic fruits (Figure 6(a)) is correlated with an increase in ethylene synthesis (Figure 4(a)).

Due to the economic importance of fruit softening, we measured three important hydrolyses that are implicated in tomato cell wall degradation. The activity of polygalacturonase was found to be very low in unripe tomato fruits (Figure 6(b)), but as fruit started to soften, there was a dramatic increase in its activity upon storage. The activity reached a maximum value after 10 days at ambient temperature in wildtype fruit, whereas the maximum was reached after 32 days in transgenic fruit. PME activity was detected in high levels in mature green fruits. The enzyme activity increased very gradually during ripening in transgenic fruits (Figure 6(c)). The increase was more than 60% in wildtype fruits and approximately 40% in transgenic fruits. In fruits of wildtype tomato, the values of *β*-gal activity were increased during the first 6 days of storage, and then remain constant to day 10 (Figure 6(d)). The increase in enzyme activity was negligible in RNAi fruits. They reached similar levels of 3.36 nkat·g^−1^ at the end of storage. As the results showed, *β*-galactosidase activity was higher in wildtype fruits compared to transgenic fruits, but according to the recorded data, the difference was not significant (*P* >0.05).

During ripening, softening of fruit is caused by the conversion of protopectin into soluble polyuronides [41]. This tightly bound protopectin is degraded into soluble pectins, which are found loosely bound to the cell walls. This phenomenon is attributed to textural softening during ripening [42]. In present research, transgenic tomato fruits carrying RNAi *ACO1* gene, retained firmness for longer periods of time than the wildtype ones (Figure 6(a)). As Figures 6(a), 6(b), and 6(c) show, there is a correlation between firmness loss and PG and PME activity. In transgenic fruits, slow rate of increasing the activity of enzyme resulted in slowing the softening process of the fruits in comparison with wildtype. High pectin demethylesterification activities, as catalyzed by PME, are required not only for subsequent PG action but also to modify pH and cation exchange properties of the wall, which might, in turn, affect activity of other wall degrading enzymes [43]. According to present data, no significant differences were recorded during storage conditions in *β*-gal activity, which suggests that *β*-galactosidase may not be involved in the softening of MT1 tomato during ripening. These findings showed no increase in total *β*-galactosidase activity during ripening of tomatoes, which seems to be typical for tomato fruit species [40]. Other glycanase enzymes such as (1 → 4)-*β*-glucanases with *β*-galactanase activities may have significant impact on wall disassembly during ripening, which needs to be studied more in this variety of tomato.

## 4. Conclusion

The work presented here has successfully demonstrated that by using RNAi technology, several transgenic lines of lowland tomato cultivar MT1, harboring an hpRNA-ACO1 construct, showed lower ethylene production because the transgenic fruits displayed delayed postharvest life with no phenotypic changes and similar amounts of SSC, TA and ascorbic acid as compared to wildtype fruits. Thus, hpRNAi ACO1 could effectively be used to delay postharvest damage, especially in climacteric fruits.

## Figures and Tables

**Table 1:** a* value of tomato fruits.

Fruits	Days								
	a* value								
	0	4	8	12	16	20	24	28	32
Wild-type	−17.02	13.49	30.59	38.63	nd	nd	nd	nd	nd
	pq	h	c	a					
RNAi	−17.16	−16.29	−13.31	−5.19	11.32	21.59	27.91	30.97	37.42
	qr	p	mn	k	hi	de	cd	bc	ab

Means with the same letters in a column are not significantly different at *P* ≤ 0.05 using LSD. Each value is the mean of four replicates.

nd: not detected due to rotting.

## References

[1] Bapat VA, Trivedi PK, Ghosh A, Sane VA, Ganapathi TR, Nath P (2010). Ripening of fleshy fruit: molecular insight and the role of ethylene. *Biotechnology Advances*.

[2] Stearns JC, Glick BR (2003). Transgenic plants with altered ethylene biosynthesis or perception. *Biotechnology Advances*.

[3] Oeller PW, Min-Wong L, Taylor LP, Pike DA, Theologis A (1991). Reversible inhibition of tomato fruit senescence by antisense RNA. *Science*.

[4] Hamilton AJ, Lycett GW, Grierson D (1990). Antisense gene that inhibits synthesis of the hormone ethylene in transgenic plants. *Nature*.

[5] Ayub R, Guis M, Amor MB (1996). Expression of ACC oxidase antisense gene inhibits ripening of cantaloupe melon fruits. *Nature Biotechnology*.

[6] Henzi MX, Christey MC, McNeil DL, Davies KM (1999). Agrobacterium rhizogenes-mediated transformation of broccoli (*Brassica oleracea* L. var. *italica*) with an antisense 1-aminocyclopropane-1-carboxylic acid oxidase gene. *Plant Science*.

[7] Aida R, Shibata M (1998). Developmentally regulated transgene silencing in Torenia. *Breeding Science*.

[8] Peters JA, Schuch MW, Silva JA, Rombaldi CV (1999). Transformação genética do meloeiro e da macieira. *Biotecnologia Cîencia e Desenvolvimento*.

[9] Klee HJ, Hayford MB, Kretzmer KA, Barry GF, Kishore GM (1991). Control of ethylene synthesis by expression of a bacterial enzyme in transgenic tomato plants. *Plant Cell*.

[10] Good X, Kellogg JA, Wagoner W, Langhoff D, Matsumura W, Bestwick RK (1994). Reduced ethylene synthesis by transgenic tomatoes expresing S-adenosylmethionine hydrolase. *Plant Molecular Biology*.

[11] Helliwell C, Waterhouse P (2003). Constructs and methods for high-throughput gene silencing in plants. *Methods*.

[12] Helliwell CA, Varsha Wesley S, Wielopolska AJ, Waterhouse PM (2002). High-throughput vectors for efficient gene silencing in plants. *Functional Plant Biology*.

[13] Xiong AS, Yao QH, Peng RH, Li X, Han PL, Fan HQ (2005). Different effects on ACC oxidase gene silencing triggered by RNA interference in transgenic tomato. *Plant Cell Reports*.

[14] Wang A, Li J, Zhang B, Xu X, Bewley JD (2009). Expression and location of endo-*β*-mannanase during the ripening of tomato fruit, and the relationship between its activity and softening. *Journal of Plant Physiology*.

[15] Meli VS, Ghosh S, Prabha TN, Chakraborty N, Chakraborty S, Datta A (2010). Enhancement of fruit shelf life by suppressing N-glycan processing enzymes. *Proceedings of the National Academy of Sciences of the United States of America*.

[16] Lopez-Gomez R, Gomez-lim MA (1992). A method for extracting intact RNA from fruit rich in polysaccharides using ripe mango mesocarp. *Hort Science*.

[17] (2004). Technology with Clonase TM II Version A, A universal technology to clone DNA sequences for functional analysis and expression in multiple systems.

[18] Ling HQ, Kriseleit D, Ganal MW (1998). Effect of ticarcillin/potassium clavulanate on callus growth and shoot regeneration in Agrobacterium-mediated transformation of tomato (*Lycopersicon esculentum* Mill.). *Plant Cell Reports*.

[19] Graham GC, Mayers P, Henry RJ (1994). A simplified method for the preparation of fungal genomic DNA for PCR and RAPD analysis. *BioTechniques*.

[20] Sambrook J, Fritsch EF, Maniatis T (1989). Molecular cloning. *A Laboratory Manual*.

[21] Chomczynski P, Mackey K (1995). Modification of the TRI Reagent(TM) procedure for isolation of RNA from polysaccharide- and proteoglycan-rich sources. *BioTechniques*.

[22] Hoeberichts FA, Van Der Plas LHW, Woltering EJ (2002). Ethylene perception is required for the expression of tomato ripening-related genes and associated physiological changes even at advanced stages of ripening. *Postharvest Biology and Technology*.

[23] Ranganna S (1977). *Manual of Analysis of Fruit and Vegetable Products*.

[24] Ali ZM, Chin LH, Lazan H (2004). A comparative study on wall degrading enzymes, pectin modifications and softening during ripening of selected tropical fruits. *Plant Science*.

[25] Freed RD, Scott DE (1986). *MSTAT-C Crop and Soil Science Department*.

[26] Chase W, Brown F (1997). *General statistics*.

[27] Dolan L (1996). Pattern in the root epidermis: an interplay of diffusible signals and cellular geometry. *Annals of Botany*.

[28] Picton S, Barton SL, Bouzayen M, Hamilton AJ, Grierson D (1993). Altered fruit ripening and leaf senescence in tomatoes expressing an antisense ethylene-forming enzyme transgene. *Plant Journal*.

[29] Schaffer RJ, Friel EN, Souleyre EJF (2007). A genomics approach reveals that aroma production in apple is controlled by ethylene predominantly at the final step in each biosynthetic pathway. *Plant Physiology*.

[30] Bolitho KM, Lay-Yee M, Knighton ML, Ross GS (1997). Antisense apple ACC-oxidase RNA reduces ethylene production in transgenic tomato fruit. *Plant Science*.

[31] Xie Y, Zhu B, Yang X (2006). Delay of postharvest ripening and senescence of tomato fruit through virus-induced LeACS2 gene silencing. *Postharvest Biology and Technology*.

[32] Alexander L, Grierson D (2002). Ethylene biosynthesis and action in tomato: a model for climacteric fruit ripening. *Journal of Experimental Botany*.

[33] Anjanasree KN, Verma PK, Bansal KC (2005). Differential expression of tomato ACC oxidase gene family in relation to fruit ripening. *Current Science*.

[34] Biale JB, Haard NF, Salunkhe DK (1975). Synthetic and degrading process in fruit ripening. *Postharvest Biology, Handling of Fruits and Vegetables*.

[35] Kumar A, Ghuman BS, Gupta AK (1999). Non-refrigerated storage of tomatoes—effect of HDPE film wrapping. *Journal of Food Science and Technology*.

[36] Selvaraj Y, Kumar R, Pal DK (1989). Changes in sugars, organic acids, amino acids, lipid constituents and aroma characteristics of ripening mango (*Mangifera indica* L) fruit. *Journal of Food Science and Technology*.

[37] Rohani MY, Zaipun MZ, Norhayati M (1997). Effect of modified atmosphere on the storage life and quality of Eksotika papaya. *Journal of Tropical Agriculture and Food Science*.

[38] Hall CB (1968). Changes in titratable acidity of tomato fruits subjected to low temperatures. *Hort Science*.

[39] Mathooko FM (2003). A comparative study of the response of tomato fruit to low temperature storage and modified atmosphere packaging. *African Journal of Food, Agriculture, Nutrition and Development*.

[40] Ali A, Maqbool M, Ramachandran S, Alderson PG (2010). Gum arabic as a novel edible coating for enhancing shelf-life and improving postharvest quality of tomato (*Solanum lycopersicum* L.) fruit. *Postharvest Biology and Technology*.

[41] John MA, Dey PM (1986). Postharvest changes in fruit cell wall. *Advances in Food Research*.

[42] Doreyappa Gowda IN, Huddar AG (2001). Studies on ripening changes in mango (*Mangifera indica* L.) fruits. *Journal of Food Science and Technology*.

[43] Micheli F (2001). Pectin methylesterases: Cell wall enzymes with important roles in plant physiology. *Trends in Plant Science*.

